# Environmental impact of omnivorous, ovo-lacto-vegetarian, and vegan diet

**DOI:** 10.1038/s41598-017-06466-8

**Published:** 2017-07-21

**Authors:** Alice Rosi, Pedro Mena, Nicoletta Pellegrini, Silvia Turroni, Erasmo Neviani, Ilario Ferrocino, Raffaella Di Cagno, Luca Ruini, Roberto Ciati, Donato Angelino, Jane Maddock, Marco Gobbetti, Furio Brighenti, Daniele Del Rio, Francesca Scazzina

**Affiliations:** 1 Human Nutrition Unit, Department of Food and Drug, University of Parma, Parma, Italy; 2 Department of Pharmacy and Biotechnology, Alma Mater Studiorum University of Bologna, Bologna, Italy; 3 Department of Agricultural, Forest and Food Science, University of Turin, Turin, Italy; 4 Faculty of Science and Technology, Libera Università di Bolzano, Bolzano, Italy; 5 Barilla Center for Food & Nutrition, Parma, Italy; 6 MRC Human Nutrition Research, Elsie Widdowson Laboratory, Cambridge, CB1 9NL United Kingdom; 7 The Need for Nutrition Education/Innovation Programme (University of Cambridge) C/O MRC Elsie Widdowson Laboratory, Cambridge, CB1 9NL United Kingdom

## Abstract

Food and beverage consumption has a great impact on the environment, although there is a lack of information concerning the whole diet. The environmental impact of 153 Italian adults (51 omnivores, 51 ovo-lacto-vegetarians, 51 vegans) and the inter-individual variability within dietary groups were assessed in a real-life context. Food intake was monitored with a 7-d dietary record to calculate nutritional values and environmental impacts (carbon, water, and ecological footprints). The Italian Mediterranean Index was used to evaluate the nutritional quality of each diet. The omnivorous choice generated worse carbon, water and ecological footprints than other diets. No differences were found for the environmental impacts of ovo-lacto-vegetarians and vegans, which also had diets more adherent to the Mediterranean pattern. A high inter-individual variability was observed through principal component analysis, showing that some vegetarians and vegans have higher environmental impacts than those of some omnivores. Thus, regardless of the environmental benefits of plant-based diets, there is a need for thinking in terms of individual dietary habits. To our knowledge, this is the first time environmental impacts of three dietary regimens are evaluated using individual recorded dietary intakes rather than hypothetical diet or diets averaged over a population.

## Introduction

It is well known that food choices are strong determinants of human health, but recently awareness has grown about the fact that the foods and beverages we produce, choose and consume may significantly affect the environment^1–3^. Since plant-based diets often emerge as nutritionally and environmentally advantageous^4^, a potential strategy to reduce both the rate of many human non-communicable diseases and prevent environmental deterioration might lie in promoting the consumption of plant-based instead of animal foods^4–8^.

The Mediterranean Diet (MD), which could be considered a plant-oriented dietary approach, appears able to face both health and environmental concerns. A MD regimen has been associated with reduced incidence of obesity, type 2 diabetes, cardiovascular disease, and has been shown to represent a valid preventative strategy towards certain cancers^9–13^. At the same time, MD has been described as a wise choice to reduce the environmental impact associated with food consumption^3, 6, 14^.

Despite the general agreement on the environmental impact of food^1^, there is a lack of information about the real influence of the whole diet not based on hypothetical intake on different indexes of environmental impact. Although greenhouse gas emissions have been extensively studied, the assessment of other indicators, such as water consumption and land use demand, have not been examined in relation to specific populations and actual dietary choices^15^. To improve evidence-based nutritional and environmental joint recommendations, more thorough research should be carried out to properly demonstrate the exact capability of dietary regimens on overall environmental impact^5^.

The extent to which real diet affect the environment has usually been analysed by applying Life Cycle Assessment (LCA) method to single foodstuffs or food groups^16^, failing to reflect actual eating habits. Just like what happened in the framework of nutritional epidemiology, that has now leaped from a short-sighted focus on single dietary components to a more integrative approach focused on whole diets, the whole concept of environmental impact evaluation urgently needs to evolve toward a comprehensive approach^17^. LCA analysis is based mainly on dietary recommendations, hypothetical diets or average consumption of population groups^18^. However, previous studies have shown that compared with recommendations or model diets, food consumption based on recorded diets is usually associated with higher environmental impacts^19^. Therefore, using actual food intake data appears to be the best strategy to perform realistic, not theoretical, environmental evaluations^18^. In addition, using recorded dietary intake allows the exploration of more detailed associations between environmental impact factors and food groups, nutrients, and/or calories actually consumed by a person or a group of people^19, 20^.

Interdisciplinary research is needed to integrate both health and environmental variables in relation to human nutrition and, in this framework, the use and analysis of real, diets within a real-life setting should be encouraged and appears to be a crucial step for the evolution of nutrition ecology and for achieving behavioural changes^4, 17, 21^. To the best of our knowledge, no study has been so far conducted by considering, simultaneously, the three major environmental indicators of the agro-food system in the framework of different dietary regimens based on real food intakes. Previous studies only provided data for different food or food groups, meals, and dietary models, generally linked to a national population^18^, without taking into account real diets, or the variance existing within a whole population^19^. For these reasons, this study aimed (i) to explore the environmental impact of omnivorous, ovo-lacto-vegetarian, and vegan diets in a real-life context of an Italian population; (ii) to examine the inter-individual variability within each dietary group.

## Methods

### Subjects

Volunteers were recruited for a previous observational multi-centre study and were enrolled across four centres in Italy (Bari, Bologna, Parma, and Turin). A total of 153 apparently healthy adults (aged 18–60 years), recruited according to their self-reported habitual diets, completed the study: 51 omnivores (O), 51 ovo-lacto-vegetarians (VG), and 51 vegans (V). Inclusion and exclusion criteria, recruitment procedure, and characteristics of the subjects have been fully described by De Filippis and colleagues^22^.

The study protocol (registered at clinicaltrials.gov as NCT02118857 on April 18^th^, 2014) was approved by the Ethics Committees of Bari, Bologna, Parma, and Turin and carried out according to the International Conference on Harmonisation Good Clinical Practice guidelines. All the volunteers signed an informed consent at recruitment. Enrolled participants were instructed to fill in a 7-d weighed food record to obtain accurate information on food and beverages consumed the week after the visit with researchers.

### Dietary information

Participants were asked to record all food and beverages consumed over 7 consecutive days using a weighed food record, as previously described^22, 23^, and to send the completed food record to the Department of Food Science of the University of Parma. Food and drink items consumed were divided into food categories, principally to control the accuracy of the enrolment of participants in one of the three diet groups, based on self-reported eating habits. The food database of the European Institute of Oncology^24^ was used to calculate daily energy and nutrient intakes. The Italian MD Index^25^ was used to evaluate the level of adherence to the Mediterranean dietary pattern, a measure of the participants’ diet healthiness. This tool is a 11-unit dietary score specific for the Italian population and it attributes positive points to Mediterranean foods (e.g. pasta, Mediterranean vegetables, fruit, legumes, olive oil, and fish) and negative points to non-Mediterranean foods (e.g. soft drinks, butter, red meat, potatoes and alcohol). Ethanol received 1 point for intake up to 12 g/day; abstainers and persons who consumed more than 12 g/day received a 0.

### Environmental impacts

Environmental impact analysis was carried out to analyse the degree to which differences in dietary choices affect the environment by using data calculated according to the LCA methodology, which takes into consideration all phases of food chain^26^. In particular, the environmental impact database of the Barilla Centre for Food and Nutrition^27, 28^ was used to evaluate environmental impact of the three dietary groups, taking into account three indexes considered to be the most representative of the agro-food system^29^: the carbon footprint (CF), the water footprint (WF), and the ecological footprint (EF). These three indexes account for the greenhouse gas emissions, the consumption of water resources, and the amount of biologically productive land/sea needed to produce a unit of food product, respectively. The CF (expressed as g CO_2_ eq./kg), WF (L/kg), and EF (global m^2^/kg) of foodstuffs were weighed according to the actual consumption, as previously described^30^, and the mean daily CF (g CO_2_ eq./day), WF (L/day), and EF (global m^2^/day) were calculated for each participants.

### Statistics

Descriptive statistics and statistical analysis have been carried out using SPSS® statistics 21.0 software (IBM, Chicago, IL), and performed at *P* < 0.05 of significance level. Daily energy and nutrient intakes, CF, WF, and EF are presented as mean and standard deviation, as the distribution of these variables was normal.

A one-way ANOVA with *post hoc* Tukey HSD test was employed to assess energy and nutrient intake differences among the three diet groups, and to evaluate the effects of each dietary approach, also by food groups, on CF, WF, and EF. Food group intakes and Italian MD score are presented as medians and interquartile range, as the distribution of these variables was invariably skewed from normality. To assess the differences in food group intakes and in the Italian MD score among the three dietary approaches, non-parametric Kruskal-Wallis test was performed, and, when significant, the pairwise multiple comparison was applied to define specific differences.

Principal component analysis (PCA) was applied to achieve a better understanding of the characteristics of each diet group and to explore the inter-individual variability. In order to simplify the model, PCA was carried out considering energy and nutrient intakes, adherence to the MD pattern, and environmental impact indicators, without keeping into account food groups. PCA was carried out with varimax rotation to explore the relationship between environmental impacts and dietary factors in each dietary group. The same configuration was used to perform a second PCA, considering nutrient intakes, adherence to the MD, and environmental impact indicators adjusted by the energy intake. One-way ANOVA with Tukey HSD test was employed to evaluate differences in the scores of each principal component (PC) among dietary groups, for both PCAs.

## Results

One hundred and fifty-three 7-d food records were collected. Participants belonging to the different diet groups were similar in terms of BMI, age, and gender (Table 1). Daily intakes of food and beverages, grouped in the same categories for the environmental impact analysis, and Italian MD score, as measured in the three diet groups, are reported in Table 2. As expected, VG and V were the highest consumers of vegetables and fruits (678.7 and 871.4 g/d, respectively), whereas O were the lowest (386.1 g/d). On the other hand, foods of animal origin (meat, fish, milk and dairy products, eggs, and animal fat) were significantly higher for O (357.3 g/d compared with 83.2 g/d for VG and as expected 0.0 g/d for V). The three dietary groups showed a medium to high level of adherence to the MD^25^, and MD score was significantly higher in the V group (7.0) and lower for the O group (4.0).

**Table 1:** Characteristics of the participants for each of the three diet groups.

Characteristics	Diet type		
	O	VG	V
	N = 51	N = 51	N = 51
BMI (kg/m^2^)	22.1 ± 2.0	21.9 ± 2.5	21.3 ± 2.2
Age (years)	37 ± 9	39 ± 9	37 ± 10
Gender (M/F)	23/28	18/33	23/28

Values are mean ± standard deviation of fifty-one independent measurements. N, total number; M, male; F, female.

**Table 2:** Daily food group intakes assessed by the 7-d food record and Italian Mediterranean Index for each of the three diet groups.

Dietary information	Diet type		
	O	VG	V
	N = 51	N = 51	N = 51
Coffee and tea (mL/d)	157.1 (121.4)^ab^	207.1 (231.4)^a^	107.1 (165.6)^b^
Alcoholic beverages (mL/d)	121.4 (219.6)^a^	51.0 (131.2)^ab^	47.1 (140.4)^b^
Soft drinks (mL/d)	0.0 (64.3)^a^	0.0 (0.0)^b^	0.0 (0.0)^b^
Fruit juices (mL/d)	28.6 (57.1)^a^	6.1 (55.0)^a^	28.6 (106.8)^a^
Meat and meat products (g/d)	113.6 (68.6)^a^	0.0 (0.0)^b^	0.0 (0.0)^b^
Fish (g/d)	34.3 (45.7)^a^	0.0 (0.0)^b^	0.0 (0.0)^b^
Eggs (g/d)	15.0 (22.5)^a^	13.2 (22.2)^a^	0.0 (0.0)^b^
Milk and dairy products (g/d)	188.6 (174.5)^a^	68.6 (140.1)^b^	0.0 (0.0)^c^
Vegetable alternatives (g/d)	0.0 (0.0)^b^	77.9 (171.7)^a^	150.7 (241.3)^a^
Animal fat (g/d)	5.9 (11.4)^a^	1.4 (4.3)^b^	0.0 (0.0)^c^
Vegetable fat (g/d)	35.71 (15.93)^c^	44.29 (17.97)^b^	56.47 (21.17)^a^
Cereals and derivatives (g/d)	246.9 (81.3)^b^	250.4 (99.8)^ab^	299.3 (107.8)^a^
Potatoes and other tubers (g/d)	40.0 (40.0)^a^	42.9 (45.8)^a^	40.0 (51.1)^a^
Legumes (g/d)	18.6 (34.0)^b^	50.7 (53.9)^a^	64.3 (74.3)^a^
Vegetables (g/d)	219.0 (117.1)^b^	407.6 (229.4)^a^	478.5 (238.7)^a^
Fruit (g/d)	167.1 (156.4)^b^	271.1 (180.9)^a^	392.9 (420.4)^a^
Nuts and dried fruits (g/d)	1.6 (7.1)^b^	8.6 (17.9)^a^	11.6 (24.1)^a^
Sweets and desserts (g/d)	116.4 (64.0)^a^	86.2 (52.9)^a^	26.9 (26.4)^b^
Italian MD Index	4.0 (3.0)^c^	6.0 (2.0)^b^	7.0 (2.0)^a^

Values are median (interquartile range) of fifty-one independent measurements. Different letters indicate significantly different values (*P* < 0.05) as calculated by non-parametric Kruskal-Wallis test with pairwise multiple comparisons. MD, Mediterranean diet; O, omnivores; VG, ovo-lacto-vegetarians; V, vegans.

With respect to energy and macronutrient intake (Table 3), although energy intake was similar among the three diet groups, protein and carbohydrate intakes in O subjects were the highest and the lowest, respectively, while V and VG groups showed similar intakes. The highest fat intake was seen in the omnivores, while the lowest characterised vegans.

**Table 3:** Daily energy and nutrient intakes (as average of 7-d food record) for each of the three diet groups.

Nutritional information	Diet type		
	O	VG	V
	N = 51	N = 51	N = 51
Total energy (kcal/d)	2471.3 ± 366.4^a^	2392.7 ± 314.3^a^	2325.7 ± 324.3^a^
Total carbohydrates (g/d)	284.1 ± 63.2^b^	315.8 ± 51.8^a^	337.4 ± 58.3^a^
Energy from carbohydrates (%)	46	53	58
Total fat (g/d)	107.7 ± 18.2^a^	95.8 ± 18.3^b^	81.6 ± 21.0^c^
Energy from fat (%)	39	36	32
Total protein (g/d)	91.5 ± 15.9^a^	74.5 ± 22.2^b^	69.9 ± 16.7^b^
Energy from protein (%)	15	12	12

Values are mean ± standard deviation of fifty-one independent measurements. Different letters indicate significantly different values (*P* < 0.05) as calculated by one-way ANOVA with *post hoc* Tukey HSD test among the three diet groups. O, omnivores; VG, ovo-lacto-vegetarians; V, vegans.

**Figure 1:**
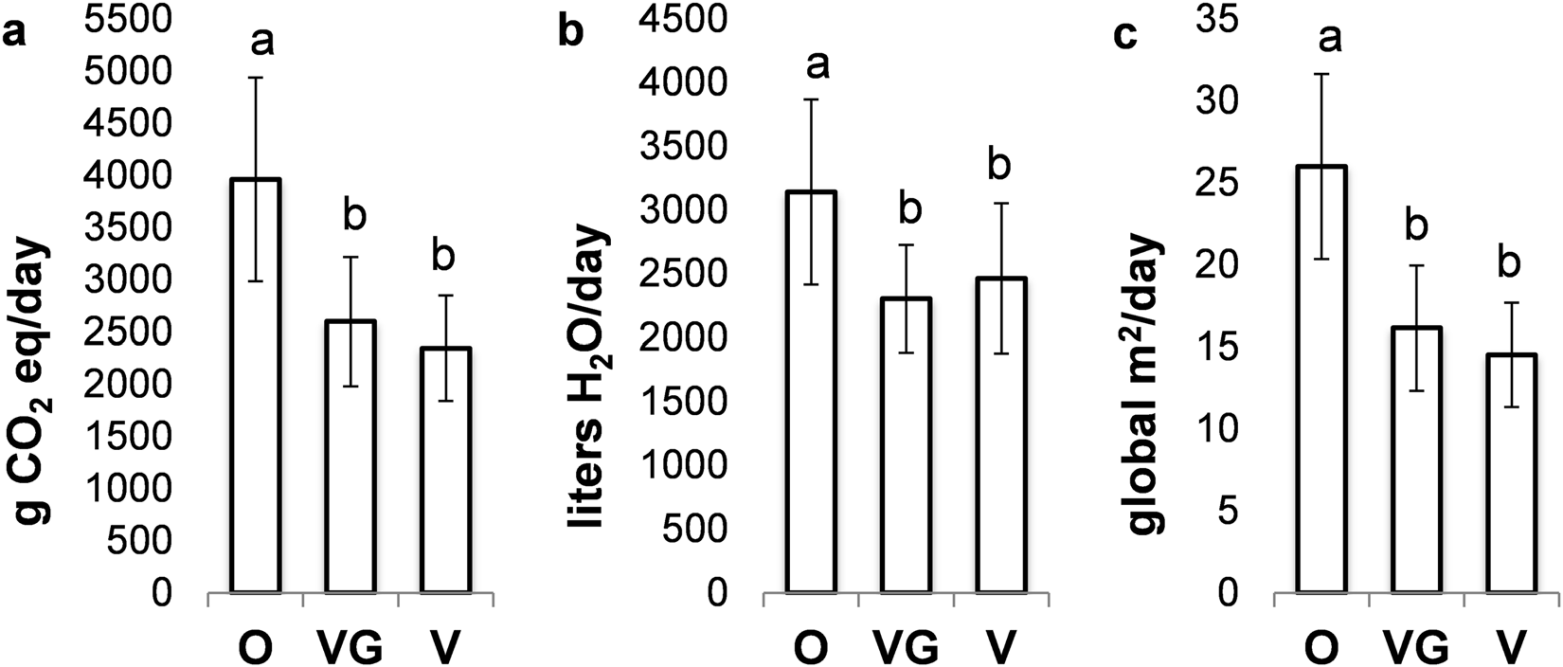
Environmental footprints: Daily carbon (**a**), water (**b**), and ecological (**c**) impacts expressed as average of 7-d food records (grams of CO_2_ equivalent/d, litres of H_2_O/d, and square meters of land/d, respectively). Values are means ± standard deviation of fifty-one independent measurements for each diet group. Different letters indicate significantly different values (*P* < 0.001) as calculated by one-way ANOVA with *post hoc* Tukey HSD test among the three diet groups. O, omnivores; VG, ovo-lacto-vegetarians; V, vegans.

The analysis of the environmental impacts of the three diet groups revealed how the animal-based diet is considerably associated with a higher impact for each environmental indicator evaluated. Indeed, the O choice generated significantly worse CF, WF, and EF (*P* < 0.001) when compared to the other diets (Fig. 1). No differences were found regarding the environmental impact when comparing VG and V groups.

The contribution of each food group on all the three environmental indicators by diet groups is presented in Table 4. Although food of animal origin was not the category consumed in greatest quantity, it was the largest contribution to footprint values for the O group. Meat, fish, and other animal-based foods (i.e. eggs, milk and dairy products, and animal fat) made the largest contribution to the environmental footprints of the O group, while animal-based foodstuffs had an intermediate impact for the VG one. As expected, foods of animal origin were not represented in the V group. The contribution of the meat and fish category to the omnivorous diet was: 37% for the CF, 38% for the WF, and 44% for the EF. Other animal-based foodstuffs contributed by 22% and 26% to the CF, and by 26% and 31% to the EF of the O and VG groups, respectively, whereas they represented the 17% of the WF for both the O and VG groups. Conversely, the greatest part of the environmental impact of V and VG was attributable to the consumption of vegetable foods. The sum of the food categories “cereals and their derivatives” and “other vegetable-based foods” (e.g. vegetables, fruits, legumes, nuts and dried fruit, potatoes and other tubers, vegetable fat, and vegetable alternatives) accounted for 24%, 56%, and 84% of the CF, 31%, 69%, and 92% of the WF, and 21%, 58%, and 90% of the EF for the O, VG, and V groups, respectively. The smallest part of each environmental indicator was linked to sweets and desserts, and drinks.

**Table 4:** Daily carbon footprint, water footprint, and ecological footprint values of food groups (as average of 7-d food record) for each of the three diet groups.

Indicator	Food group	Diet type		
		O	VG	V
		N = 51	N = 51	N = 51
Carbon Footprint (g CO_2_ eq./d)	Drinks	430.9 ± 342.9^a^	299.2 ± 355.3^a^	325.0 ± 385.0^a^
	Meat and Fish	1447.2 ± 756.8^a^	0.0 ± 0.0^b^	0.0 ± 0.0^b^
	Other animal-based foods	901.9 ± 363.6^a^	628.9 ± 465.2^b^	0.0 ± 0.0^c^
	Cereals and their derivatives	425.5 ± 110.1^b^	490.4 ± 133.4^ab^	548.0 ± 200.7^a^
	Other vegetable-based foods	503.3 ± 170.1^c^	995.8 ± 367.5^b^	1422.5 ± 381.4^a^
	Sweets and desserts	250.8 ± 125.1^a^	184.1 ± 109.6^b^	47.0 ± 44.5^c^
	Total	3959.3 ± 975.8^a^	2598.3 ± 619.0^b^	2336.1 ± 496.8^b^
Water Footprint (L/d)	Drinks	174.3 ± 127.1^a^	129.2 ± 138.1^a^	158.1 ± 152.1^a^
	Meat and Fish	1176.9 ± 603.5^a^	0.0 ± 0.0^b^	0.0 ± 0.0^b^
	Other animal-based foods	567.9 ± 236.8^a^	385.5 ± 287.7^b^	0.0 ± 0.0^c^
	Cereals and their derivatives	330.4 ± 81.3^b^	370.2 ± 96.7^ab^	412.8 ± 147.4^a^
	Other vegetable-based foods	623.3 ± 223.9^c^	1224.2 ± 449.7^b^	1835.4 ± 676.1^a^
	Sweets and desserts	268.0 ± 132.3^a^	195.6 ± 114.7^b^	51.8 ± 47.6^c^
	Total	3140.8 ± 726.5^a^	2304.7 ± 421.6^b^	2455.0 ± 582.2^b^
Ecological Footprint (global m^2^/d)	Drinks	1.0 ± 0.9^a^	0.8 ± 1.0^a^	1.1 ± 1.2^a^
	Meat and Fish	11.2 ± 4.7^a^	0.0 ± 0.0^b^	0.0 ± 0.0^b^
	Other animal-based foods	7.0 ± 2.9^a^	4.7 ± 3.6^b^	0.0 ± 0.0^c^
	Cereals and their derivatives	2.5 ± 0.6^b^	2.8 ± 0.7^ab^	3.1 ± 1.1^a^
	Other vegetable-based foods	2.7 ± 1.4^c^	6.6 ± 2.9^b^	10.0 ± 2.9^a^
	Sweets and desserts	1.6 ± 0.8^a^	1.2 ± 0.7^b^	0.3 ± 0.3^c^
	Total	26.0 ± 5.6^a^	16.1 ± 3.8^b^	14.5 ± 3.1^b^

Values are mean ± standard deviation of fifty-one independent measurements. Different letters indicate significantly different values (*P* < 0.05) as calculated by one-way ANOVA with *post hoc* Tukey HSD test among the three diet groups. O, omnivores; VG, ovo-lacto-vegetarians; V, vegans. Drinks: alcoholic beverages, soft drinks, and fruit juices. Meat and Fish: meat and meat products, and fish. Other animal-based foods: eggs, milk and dairy products, and animal fat. Cereals and their derivatives: cereals and their derivatives. Other vegetable-based foods: fruit, vegetables, nuts and dried fruits, legumes, potatoes and other tubers, vegetable alternatives, and vegetable fat. Sweets and desserts: sweets and desserts.

**Figure 2:**
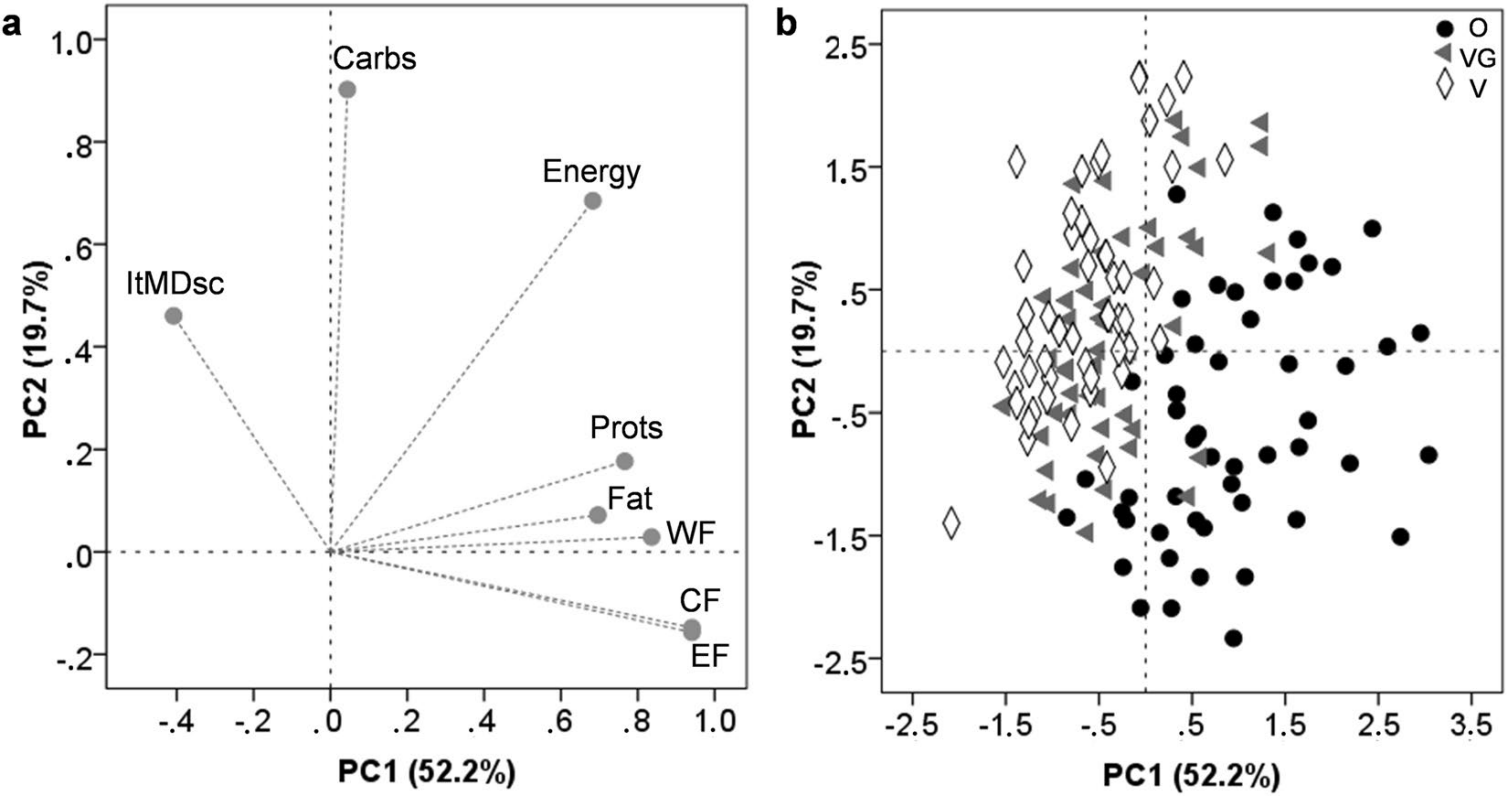
Loading plot (**a**) and score plot (**b**) obtained from the PCA with varimax of the energy and nutrient intakes, adherence to the Mediterranean dietary pattern, and environmental impact indicators for each diet group. ItMDsc, Italian Mediterranean Diet score; Energy, total energy; Carbs, total carbohydrates; Fat, total fat; Prots, total proteins; and CF, WF, and EF for carbon, water, and ecological footprint, respectively. O, omnivores; VG, ovo-lacto-vegetarians; V, vegans.

When inter-individual variability was explored by applying PCA, two PCs explained 72% of the total variation (Fig. 2a). Almost 52% of the observed variability was explained by the first PC (PC1) that correlated positively with the environmental impact indexes EF, CF, and WF, total protein intake, total fat intake, and energy intake, while negatively with the Italian MD score. Principal component 2 (PC2) had high component loadings from the total carbohydrate intake, energy intake, and MD score.

Individual scores of each PC were computed, clearly highlighting the differences existing among dietary choices (*P* < 0.001 for both PCs), as well as within each diet group (% of variability at Supplemental Table S3) (Fig. 2b). The O group, with high scores for PC1 and low scores for PC2, was defined as a dietary choice with higher CF, WF, and EF values, higher consumption of proteins and fats while lower consumption of carbohydrates, and lower adherence to the Italian MD. On the contrary, VG and V groups, which resulted to be quite similar, showed negative PC1 scores and positive PC2 scores. This implies that VG and V groups are characterized by a lower environmental impact, a lower consumption of proteins and fats, a higher consumption of carbohydrates, and a higher adherence to the Italian MD. Inter-individual variability within every diet groups was mainly affected by the energy intake and the level of intake of carbohydrates (variability within PC2 was higher than variability within PC1, Supplemental Table S3). Variability among subjects within the O group was higher than that recorded for the VG and V groups (see dispersion at Fig. 2b and standard deviations for each PC at Supplemental Table S3). Moreover, it must be taken into account that some specific subjects within a particular group may have environmental impacts notably different from the other subjects belonging to the same group. Therefore, to overcome the effect that the energy density of a diet may have on the magnitude of the environmental impact of each subject, a second PCA was performed after normalizing each variable by energy intake of each subject. Results of this new PCA confirmed the same trend for each dietary group, as well as the high inter-individual variability within every group (Supplemental Fig. S1 and Supplemental Table S4). Besides noting the higher environmental impact of some VG and V when individually compared to some O (Supplemental Fig. S1b), the presence of two vegan participants having extremely high environmental impact values was particularly interesting. When delving into their nutritional data, these two subjects were characterized by the sole consumption of fruits.

## Discussion

In order to explore the environmental impact of different dietary regimens, three dietary groups were selected: omnivorous, ovo-lacto-vegetarian, and vegan. The analysis of food patterns highlighted the definition of well-matched diet groups based on participants’ self-reported eating habits.

Conversely to previous data of environment impact obtained on hypothetical diets and meals, the present results are based on recorded intakes and have the clear advantage of being realistic. The three dietary regimens were equivalent to one another for energy content, but differed from the custom diets designed in previous studies^7, 28^. As an example, the environmental impacts of different dietary patterns (omnivorous, vegetarian, and vegan) were assessed in Italy by designing a weekly well-balanced plan, with a daily average energy intake ranging between 2100 and 2300 kcal^7^. In another study dealing with the environmental impact of omnivorous, vegetarian and vegan diets, three weekly menus were designed, and the average energy intake resulted to be a little higher than 2000 kcal/d^28^. Recorded energy intakes were indeed higher than those hypothesised before, especially for O, and showed a remarkable variance within our study groups, but all fell within the reference values for Italian healthy adults^31, 32^ with a normal BMI. It should be noted that the mean energy intake of our population was similar to a designed dietary plan of 2400 kcal in which the environmental impact of omnivorous, ovo-lacto-vegetarian and vegan diets were compared^33^. Despite these similarities on energy intake, modelling approaches referring to an average diet may produce unrealistic food combination and quantities, since eating patterns vastly differ among individuals^34^. Indeed, our three diet groups differed in their nutrient profiles. Specifically, carbohydrate intake was always matching the 45–60% of daily calories recommended for the Italian population^31, 32^, with the O and V groups close to the lower and the upper values, respectively. Total fat intake fell within the reference range of 20–35% of daily calories^31, 35^ only in the V group, while O and VG volunteers had fat intakes slightly over the recommended values. Protein intake was within the recommended 10–20% of daily calories^36^ for all the diet groups. When compared to dietary models, which are standardised to match the energy contribution for protein, lipid and carbohydrate with recommended values^7, 28^, our assessment revealed: 1) lower intakes of carbohydrates in the O and VG groups, 2) lower intakes of proteins in the VG and V groups, and 3) higher intakes of lipids in all the diet groups, especially for O and VG.

These differences between real and hypothetical diets underline the need to observe and analyse recorded dietary intakes in order to properly assess the environmental impact of dietary approaches. Only a few studies have assessed the environmental impact of actual dietary intake calculated on the basis of CF, WF, and EF simultaneously. Both Donati *et al*.^30^ and Germani *et al*.^29^ evaluated the carbon, water, and land footprints of the actual food intake of the Italian adult population, but without taking into account any differences in the dietary regimen. Data on the environmental impact of the Italian household consumption are consistent with our results for all the three environmental indicators^29^. Germani *et al*. also compared the actual Italian food consumption to a MD model, highlighting the lower value of the three indices in MD compared to the actual Italian diet^29^. Similar results were found in other studies, in which a shift of the Italian average food consumption towards a MD pattern resulted in a reduction of the food environmental impact on natural resources, especially on greenhouse gas emissions^37^ and water usage^38^. Indeed, in accordance with numerous studies, the MD emerges as a dietary regimen with beneficial environmental and health effects^14, 29, 39, 40^. Along with the nutritional profiles, the notable adherence to the MD revealed a general healthy eating pattern of our participants in all the three dietary regimens. These results are consistent with those previously reported by Benedetti *et al*.^41^ in which Italians appeared to have a moderately high level of adherence to the MD.

In addition to the MD, other predominantly plant-based diets appear environmentally better than meat-based ones^21, 42, 43^. Our results are consistent with those reports, since, in the present study, VG and V diets represent a clear environmental advantage with respect to the O one for all the three environmental evaluated indicators. This aspect, which is related to the biggest environmental impact generated by a greater consumption of animal products, had already been hypothesized^7, 28^.

However, the big differences highlighted when observing virtual scenarios are not so evident when taking into account real-life contexts. In particular, the V approach was not associated with significantly lower environmental footprints when compared to VG one. A likely explanation might be that, while unprocessed plant-based foodstuffs usually replace animal-based products in hypothetical vegetarian and vegan diets^18^, the real plant-based diets are instead characterised by industrially highly-processed plant-based meat and dairy substitutes (e.g. seitan burger and soy yoghurt). Some people opt for highly processed, high-fat products instead of nutritious plant-based foodstuffs^44^. Moreover, the lower energy density of plant-based foodstuffs results in a higher food intake for V with respect to VG (around 12.5% in terms of food weight), possibly explaining the lack of environmental benefits of a vegan diet in comparison with an ovo-lacto-vegetarian choice.

Comparison of the environmental impact of different populations remains very difficult, as data found in the literature vary widely among studies. Differences in geographical areas, data sources, and dietary assessment strategies could represent explanatory factors for this variability^45^. In the present study, as an example, CF equalled 3.96 ± 0.98, 2.60 ± 0.62, and 2.34 ± 0.50 kg CO_2_-eq per person/d for the O, VG, and V group, respectively, as assessed with a 7-d food record. Without considering the specific diet of the participants, the same parameter was 3.44 kg CO_2_-eq per person/d in the Italian INRAN-SCAI cohort (2313 subjects, aged 18–65 years) using a self-recorded 3-d dietary record^29^. Differences among European populations were detected in various studies resulting in: 3.24 kg CO_2_-eq per person/d for the UK-NDNS cohort (1491 subjects, aged 19–94 years), assessed through a 4-d food record^46^; 3.55 and 4.69 kg CO_2_-eq per person/d for women and men, respectively, of the INCA2 French cohort (2624 adults) recording a 7-d food record^47^; 3.87 kg CO_2_-eq per person/d for the Nederland EPIC cohort (40011 adults), measured through a food frequency questionnaire^48^; and 3.76 and 5.04 kg CO_2_-eq per person/d, if evaluated with a food frequency questionnaire or with a 7-d weighed dietary record, respectively, in 166 Sweden volunteers^49^.

Beyond the impact of dietary choices, variations in the average environmental impact of a particular diet or a population have been attributed to differences on individual choices^50^, gender^51^ or household structure^52^. In the present study, the high inter-individual variability registered within each dietary group is worth attention. In particular, it is interesting to note the presence of some VG and V subjects with individual values of environmental impact higher than some of the O study group. The high impact of these specific subjects was mainly attributed to a high consumption of proteins and fats, but energy intake also contributed. High meat consumption and high-fat vegetarian diets were hypothesized to require more land resources than other food choices^53^. Inter-individual variability was also conditioned by energy intake of each subject, which is associated with adequate nutritional needs and influenced by energy density of the diet. When impact data were adjusted by energy intake, as also reported by Soret *et al*.^44^, the analysis brought to light an extremely high environmental impact of some subjects, in particular fruitarians.

A focus on a specific population, even though participants belong to four geographically distant areas in Italy, and the fact that seasonality, farming and cattle rearing typology, and food production methods were not taken into account for the environmental impact evaluation should be considered the main weaknesses of this study. In addition, the environmental database used presents some intrinsic limitations. Although it is regularly updated and based entirely on public data and information from scientific literature, it cannot be considered complete. As a consequence, data related to food subgroups and not to the single food item were used. Correspondence among specific foodstuffs and the subgroup to which these foods were assigned, as well as the environmental impact values for each food subgroup, are provided at Supplemental Tables S1 and S2, respectively. The WF of fish and seafood were not available and the environmental data on coffee and tea were not easily attributable because linked to dry powder and not to ready-to-use products. Nevertheless, this is the first study to evaluate simultaneously the carbon, water, and land impact of real recorded and weighed food intakes in groups, although not large, of O, VG, and V.

To reach an environmentally sustainable solution, animal-based foodstuffs should be partially replaced with fruits, vegetables, legumes, and cereals, according to nutritional guidelines. At the same time, observations regarding inter-individual variability are of critical importance as the generic definition of every dietary pattern may dramatically conceal the impact of individual choices on environmental footprints. This emphasizes the need for thinking not only in terms of dietary group but also of individual dietary habits, irrespective of dietary choice. These aspects should be considered before launching equivocal messages to the general population through the media, and/or underpinning certain dietary choices.

Dietary recommendations in terms of environmental impacts deserve further studies, considering, for instance, the choice of locally grown and seasonal products as well as agricultural and processing techniques. Health status should be also included in this multifactorial scenario dealing with food choice, energy intake, and environmental impact. This holistic approach, merging health preservation and disease prevention with environmental sustainability in the dietary habits framework should be pursued. Educating people to make little changes in their dietary behaviours could be a key action towards this common goal.

## Electronic supplementary material

Supplementary Information

Supplementary Table S1 and S2
