# Supplementary material for: Environmental impact of omnivorous, ovo-lacto-vegetarian, and vegan diet
Source: Sci Rep. 2017 Jul 21;7:6105. doi: 10.1038/s41598-017-06466-8 (PMC5522483; doi:10.1038/s41598-017-06466-8)
Supplement: Supplementary file 1 — Supplementary Information [file 41598_2017_6466_MOESM1_ESM.pdf]

Environmental impact of omnivorous, ovo-lacto-vegetarian, and vegan diet

Alice Rosi[1], Pedro Mena[1], Nicoletta Pellegrini[1]*, Silvia Turroni[2], Erasmo Neviani[1], Ilario Ferrocino[3], Raffaella Di Cagno[4], Luca Ruini[5], Roberto Ciati[5], Donato Angelino[1], Jane Maddock[6,7], Marco Gobbetti[4], Furio Brighenti[1], Daniele Del Rio[1], Francesca Scazzina[1]

Supplementary information

Supplemental Figure S1: *Loading plot (loadings -a- and scores -b-) obtained from the PCA with varimax of the nutrient intakes, adherence to the Mediterranean dietary pattern, and environmental impact indicators for each diet group normalized by energy intake*. ItMDsc, Italian Mediterranean Diet score; Carbs, total carbohydrates; Fat, total fat; Prots, total proteins; and CF, WF, and EF for Carbon, Water, and Ecological Footprint, respectively. O, omnivores; VG, ovo-lacto-vegetarians; V, vegans.

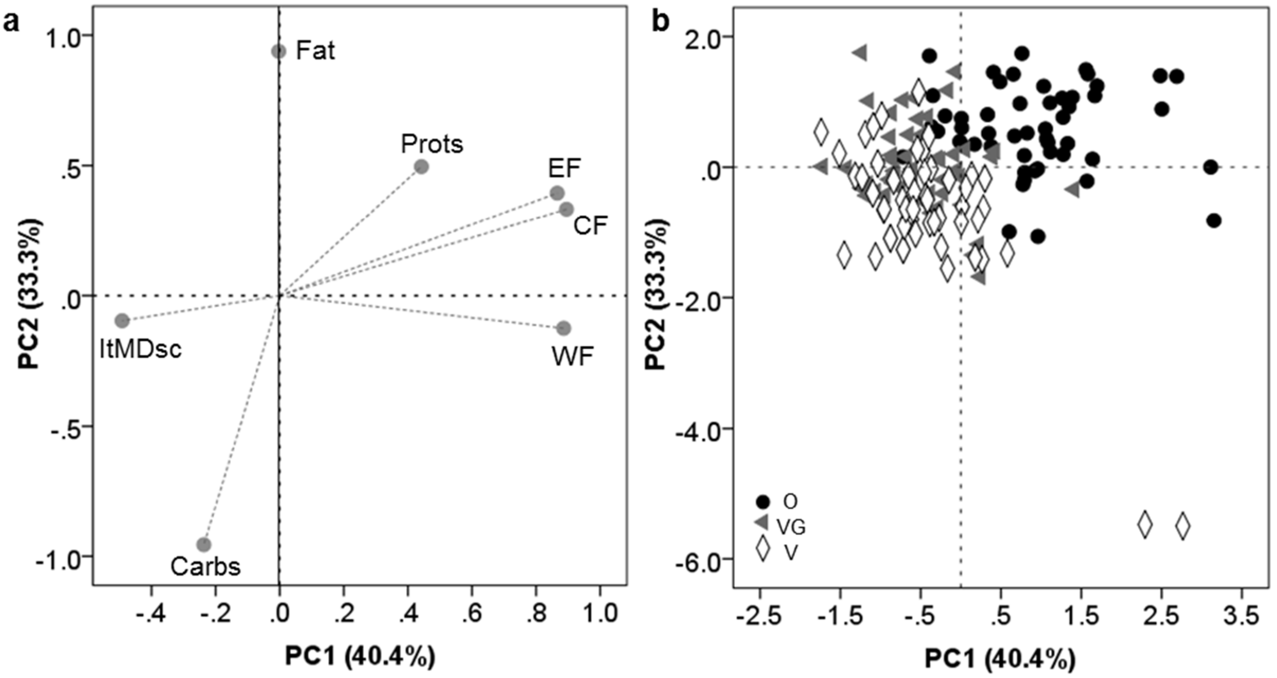

Supplemental Table S1*:* List of food items and the specific food subgroup to which every food item was assigned.

Supplemental Table S2*:* Environmental impact values (carbon, water, and ecological footprint) for each food subgroups.

Supplemental Table S3. Principal component scores for each diet group obtained from Figure 3.

Principal component (PC) scores	Diet type		
	O<br>N=51	VG<br>N=51	V<br>N=51
PC1	0.95 ± 0.93[a]	-0.32 ± 0.65[b]	-0.63 ± 0.58[b]
PC2	-0.59 ± 0.94[b]	0.14 ± 0.88[a]	0.46 ± 0.88[a]
Variability within PC1 (%)	97.89	203.13	92.06
Variability within PC2 (%)	159.32	628.57	191.30

Values are mean ± standard deviation (SD) of fifty-one independent measurements. Variability (%) within every PC was calculated as the coefficient of variation (absolute value of the ratio SD/mean*100). Different letters indicate significantly different values ($P < 0.05$) as calculated by one-way ANOVA with *post hoc* Tukey HSD test among the three diet groups. O, omnivores; VG, ovo-lacto-vegetarians; V, vegans.

Supplemental Table S4. Principal component scores for each diet group obtained from Supplemental Figure S1.

Principal component (PC) scores	Diet type		
	O<br>N=51	VG<br>N=51	V<br>N=51
PC1	0.94 ± 0.86[a]	-0.51 ± 0.55[b]	-0.43 ± 0.79[b]
PC2	0.59 ± 0.66[a]	0.06 ± 0.66[b]	-0.65 ± 1.17[c]
Variability within PC1 (%)	91.49	107.84	92.06
Variability within PC2 (%)	111.86	1112.30	178.38

Values are mean ± standard deviation (SD) of fifty-one independent measurements. Variability (%) within every PC was calculated as the coefficient of variation (absolute value of the ratio SD/mean*100). Different letters indicate significantly different values ($P < 0.05$) as calculated by one-way ANOVA with *post hoc* Tukey HSD test among the three diet groups. O, omnivores; VG, ovo-lacto-vegetarians; V, vegans.
